# Linking soil microbial community dynamics to straw-carbon distribution in soil organic carbon

**DOI:** 10.1038/s41598-020-62198-2

**Published:** 2020-03-26

**Authors:** Yao Su, Zhenchao He, Yanhua Yang, Shengqiang Jia, Man Yu, Xijing Chen, Alin Shen

**Affiliations:** 1 Institute of Environment, Resource, Soil and Fertilizer, Zhejiang Academy of Agricultural Sciences, Hangzhou, 310021 China; 2 College of Environment and Resources, Zhejiang A & F University, Hangzhou, 311300 China

**Keywords:** Soil microbiology, Carbon cycle

## Abstract

Returning crop residues is a possible practice for balancing soil carbon (C) loss. The turnover rate of organic C from crop residues to soil C is dependent on soil microbial community dynamics. However, the relationship between any temporal changes in the soil microbial community after crop straw inputs and the dynamics of straw-C distribution in the soil organic carbon (SOC) pool remains unclear. The present study investigated the allocation of straw-C into soil dissolved organic carbon (DOC), microbial biomass carbon (MBC), particulate organic carbon (POC) and mineral-associated organic carbon (MaOC) using stable isotope probing, as well as the temporal changes in the soil bacterial and fungal communities using high-throughput sequencing. After the first 180 days of straw decomposition, approximately 3.93% and 19.82% of straw-C was transformed into soil MaOC and POC, respectively, while 0.02% and 2.25% of straw-C was transformed into soil DOC and MBC, respectively. The temporal change of the soil microbial community was positively correlated with the dynamics of straw-C distribution to SOC (R > 0.5, *P* < 0.05). The copiotrophic bacteria (e.g., *Streptomyces*, *Massilia* and *Sphingobacterium*), cellulolytic bacteria and fungi (e.g., *Dyella* and *Fusarium*, *Talaromyces)*, acidophilic bacteria (e.g., *Edaphobacter* and unclassified *Acidobacteriaceae*), denitrifying and N-fixing microbes (e.g., *Burkholderia-Paraburkholderia*, *Paraphaeosphaeria* and *Bradyrhizobium)*, and fungi unclassified *Sordariomycetes* were significantly correlated with straw-C distribution to specific SOC fractions (*P* < 0.05), which explained more than 90% of the variation of straw-C allocation into soils. Copiotrophic, certain cellulolytic and denitrifying microbes had positively correlated with DOC- and MaOC-derived from straw, and other cellulolytic fungi (e.g., *Talaromyces*) and specific bacteria (e.g. *Bradyrhizobium*) were positively correlated with POC-derived from straw. Our results highlight that the temporal change of soil microbial community structure well reflects the conversion and distribution process of straw-C to SOC fractions.

## Introduction

Soil organic matter, the largest terrestrial carbon (C) pool, plays an important role in improving soil fertility and sustaining soil productivity^1,2^. Many agricultural management practices, such as fertilizer application, straw return, and tillage, could affect the soil organic carbon (SOC) pool^3–6^, especially the labile organic carbon fractions, such as dissolved organic carbon (DOC), microbial biomass carbon (MBC) and particulate organic carbon (POC). DOC and MBC, considered to be soil active organic C, have been reported as important factors in impacting soil fertility^7,8^. Moreover, the formation, migration and transformation of DOC can affect soil microbial community structure and activity^9–11^, and the formation of mineral-associated organic carbon (MaOC)^12,13^, which usually represents the largest fraction of SOC pool^14^. POC has been considered as a transition state of stable organic C, which is predominantly of plant origin and has a higher inherent biochemical recalcitrance than MaOC^15,16^.

Returning crop straw is a practice that has been applied widely in China as an attempt to balance soil C losses^17,18^. Most of the crop residue-derived C will be mineralized to CO_2_, and the remaining C can be transformed to SOC fractions^19^. The process of residue C conversion and distribution significantly affects SOC fraction dynamics. Williams *et al*.^20^ studied the distribution and fate of ^13^C-labeled straw residues from clover and ryegrass in soil and observed that about 20–30% of soil MBC was derived from straw-C during the incubation. Pei *et al*.^21^ reported that soil MBC contained 16.6–62.8% of maize straw-C during 365 days after ^13^C-labled maize straw inputs to different types of soil. Li *et al*.^22^ reported that the contribution of residue C to soil POC was 63% on average on day 60 and that to MaOC was 23%. Recently, it is proposed that MaOC is predominantly formed in “*in vivo* microbial turnover” pathway occurring in the early stages of decomposition from residues^12,13^. These studies suggest the importance of the interactions between straw-C distribution and SOC fractions, as well as the important role of soil microbes in straw-C conversion.

Most previous studies have focused on the succession of the soil microbial community associated with returned crop residues, which have consistently shown that the input of crop residues greatly stimulated copiotrophic bacteria, such as β- and γ-Proteobacteria, Actinobacteria, Bacteroidetes, and Firmicutes^23–26^, and fungi *Mortierellaceae*^27^ and *Fusarium*^26,28^, during the initial phase of residue decomposition. In the later phases of decomposition, the growth of oligotrophic bacteria, such as Acidobacteria, Chloroflexi, and Gemmatimonadetes, was enhanced^28,29^. However, these studies ignored the relationship between soil microbial communities and straw-C conversion and distribution. Guo *et al*.^30^ assessed the relationship between top soil bacterial communities and SOC and showed that *Burkholderia*, *Pseudomonas*, *Clostridium*, *Rudaea*, *Bacillus*, and *Gemmationas* explained 67.7% of the variance in SOC; thus, microbial communities might help to regulate SOC sequestration in the top soil under a residue-return system. Nevertheless, the mechanism by which soil bacteria and fungi are linked to the dynamics of straw-C conversion and partitioning during straw decomposition and their relative contributions remain unknown. Although available C transformed from straws might be a C and energy source, any temporal factors and the relationship with straw-C partitioning during straw decomposition remain unknown. With the decomposition of straw, metabolic intermediates with different carbon utilization efficiency (CUE) will be produced, like sugars, amino acid, phenolic acid and other aromatic compounds^13,31^. Such decomposition process will affect not only the soil microbial community composition, but also the C compounds available for mineral-sorption. Therefore, further investigation is needed to comprehensively understand the interactions between changes in soil bacterial and fungal communities and straw-C conversion dynamics after straw inputs.

The objective of this study was to assess the effects of crop straw inputs on soil bacterial and fungal communities and the factors affecting straw-C conversion and distribution, by conducting semi *in situ* experiments with ^13^C-labeled wheat straw and high-throughput sequencing techniques. We hypothesized that: (1) straw inputs would change the diversity and composition of soil bacterial and fungal community; (2) the significantly-different genera associated with straw inputs could have positive or negative correlation with straw-C conversion and distribution to SOC fractions. The linear discriminant analysis (LDA) coupled with effect size measurements (LEfSe) analysis were used to detect the key functional genera after straw inputs. Manteltests, redundancy analysis, and Spearman correlation analysis were applied to reveal the potential relationships between soil microbial communities and the dynamics of straw-C allocation to different SOC pools during straw decomposition.

## Materials and Methods

### Soil physiochemical analyses

Soil samples for the incubation experiments were collected from the top surface (0–20 cm) soil of the field site with rice-wheat rotation system, located in Guatianbu Village (29°54′36″N, 119°51′39″E), Hangzhou City, southeastern China, in June, 2016, after the wheat harvest. We collected a total of 12 top surface (0–20 cm) soil samples of approximately 5 kg each from 12 random points within the 1 ha field plot; soil samples were combined to create a composite soil, which was used for the initial physiochemical analysis and the microcosm incubation experiment. The soil was sandy loam with clay content of 14.3%, and had a pH of 6.02, an organic C content of 6.66 g kg^−1^, a total N content of 1.09 g kg^−1^, a DOC and MBC content of 26.64 and 357.26 mg kg^−1^, a POC and MaOC content of 2.56 and 4.08 g kg^−1^, respectively, and a δ^13^C value of −27.78‰.

### Straw ^13^C-labeling

The ^13^C-labeled wheat straw residue used in the experiments was obtained by harvesting mature wheat plants that were grown in gas-tight growth chambers exposed to ^13^CO_2_-fumigation for 15 days (15–29 April 2015) during the vegetative growth period (including the entire elongation stage). The CO_2_ concentration within the growth chambers was maintained at 360–380 ppm. The ^13^CO_2_ was generated by acidifying Na_2_^13^CO_3_ (1.0 M, 99 atom% ^13^C; Cambridge Isotope Laboratories, Tewksbury, MA, USA) with H_2_SO_4_ (0.5 M) in beakers that were placed inside the growth chambers. The growth chambers were installed in a wheat field during November 2014 to May 2015. An air-conditioning system was used to control the temperature inside the chamber to within 1 °C of the ambient temperature in the wheat field. The harvested aboveground, labeled wheat straw was dried at 60 °C for 12 h and then chopped into pieces less than 5 mm segments for storage in sealed jars. The wheat straw contained 6.38 g kg^−1^ total N and 352.9 g kg^−1^ total C, with a C/N of 55.3 and a δ^13^C value of 357.48‰.

### Incubation experiment

To create a common experimental treatment soil mixture, we thoroughly mixed approximately 100 g of air-dried soil (passed through 4-mm sieve) and 3.0 g of the chopped ^13^C-labeled wheat straw. A soil without wheat straw was prepared as the control treatment. Both experimental and control soil mixtures were adjusted to a soil moisture of 70% of water holding capacity prior to their placement in the simulated soil column (3.8 cm inner diameter, 5.5 cm outer diameter, and 15.5 cm high). Then, all soil columns were placed in the greenhouse to exclude the influence of precipitation, but the temperature and the duration of light in the greenhouse was kept to be the same as the ambient.

Three incubated soil columns were randomly and destructively sampled as replicates from each treatment on days of 7, 14, 28, 60, and 180 after the initial straw inputs. Fresh soil samples in the column were taken out and analyzed for DOC and MBC contents and their respective δ^13^C values coincident with all sampling dates. Subsamples of these soil samples were also air-dried, ground, sieved, and analyzed for SOC and POC contents and their respective δ^13^C values. Other samples were stored at −20 °C for soil DNA extraction and sequencing and qPCR analysis.

### Soil organic C measurements

SOC of the soil samples was determined by oxidation with potassium dichromate and titration with ferrous ammonium sulfate after the samples were sieved to <0.15 mm^32^. DOC was extracted according to Liang *et al*.^33^. Briefly, 10 g of fresh soil was shaken with 30 mL of distilled water for 30 min at 180 r min^−1^, and then centrifuged for 15 min at 4000 r min^−1^. Ten mL of the supernatant solution was filtered with a 0.45-mm filter film. The DOC was measured with a TOC analyzer (MultiN/C 3100, Analytik Jena AG, Jena, Germany). The remaining supernatant solution (15 mL) was freeze-dried and passed through 0.15-mm sieve for δ^13^C value measurements.

MBC was determined according to the chloroform fumigation extraction method^34^. The fumigated and non-fumigated soil samples were extracted with 40 mL of 0.5 M potassium sulfate for 30 min at 25 °C and 180 r min^−1^. Then, 10 mL of the supernatant solution was filtered with a 0.45-mm filter film and the MBC was determined as a DOC measurement, as detailed above. The remaining supernatant solutions (25 mL) from both the fumigated and nonfumigated soils were freeze-dried and passed through 0.15-mm sieve prior to δ^13^C value measurements.

POC was determined with the method by Cambardella and Elliott^35^ with some modifications. Briefly, 10.0 g of air-dried soil samples were dispersed in 30 mL of hexameta phosphate (5 g L^−1^) in a 150 mL conical flask by shaking on a reciprocating shaker for 15 h at 180 r min^−1^. The soil suspension was then poured into a 53-μm sieve and washed with distilled water to ensure separation. The particulate fraction was dried at 60 °C for 48 h and then weighed and ground to pass through a 0.15-mmsieve. The POC content was determined using 15 mL samples of the dried and sieved particulate fraction material with an elemental analyzer (VarioIsotopeCube, Elementar, Germany), and 15 mL was used to determine the δ^13^C value of the POC. MaOC content was determined by calculating SOC contents minus POC contents.

All δ^13^C signatures of the different SOC fractions and wheat straws were determined using an EA-IRMS (Elementar vario PYRO cube coupled to IsoPrime 100 Isotope Ratio Mass Spectrometer, Elementar, Germany). Carbon isotopic composition in this study is reported in δ notation (in ‰ units), relative to the international standard Pee Dee Belemnite.

### DNA extraction and Miseq sequencing analysis

DNA extraction was performed on each soil sample using Mobio Power Soil DNA Isolation Kits (Qiagen, USA) according to the manufacturer’s instruction manual. The extracted DNA was quantified using a Nanodrop ND-1000 spectrophotometer (Thermo Fisher Scientific, Waltham, MA, USA).

The V1–V3 region of the bacterial 16S rRNA gene and the ITS1 region for fungi were sequenced. The PCR reaction system and conditions, the method of purifying amplicons and selecting high-quality raw reads were followed as Su *et al*. described^36^. The taxonomy of each sequence was analyzed by RDP Classifier algorithm (http://rdp.cme.msu.edu/) against the SILVA 128 database for 16S rRNA genes and UNITE 7.0 for ITS using a confidence threshold of 70%.

Sequencing reads were deposited in the Sequence Read Archive at the NCBI under the accession number of SRR10507228 under the Project ID of PRJNA577745.

### Calculations and statistic analyses

We analyzed the variations in SOC fraction contents and the microbial community diversity indices for different treatments and sampling times using ANOVA in SPSS v20.0 software. Shapiro-Wilk tests were used to assess the equality of variances before performing ANOVAs. Multidimensional scaling (NMDS) analysis was used to compare the difference between the changes of soil microbial communities with and without straw inputs during the whole incubation. To search for the significantly-different genera induced by straw inputs during the whole incubation, we applied LEfSe analysis. Species and SOC fractions derived from straw distance matrixes were calculated using a Bray-Curtis dissimilarity method and compared to determine if there were correlations between the soil microbial community and the allocation of the straw-C. The relationship of soil microbial communities with straw-C conversion and allocation was analyzed by using redundancy analysis (RDA) with Monte Carlo permutation tests based on 999 random permutations in CANOCO 4.5 for Windows. A Spearman correlation analysis was performed to test the correlation of specific microbial species with the SOC fractions derived from straw.

The ^13^C content of the different SOC fractions was calculated as follows:

1 $${{\rm{R}}}_{13{\rm{C}}}/{{\rm{R}}}_{12{\rm{C}}}={{\rm{\delta }}}^{13}{\rm{C}}\,\ast \,0.0112372+0.0112372$$

2 $${{\rm{R}}}_{13{\rm{C}}}=\frac{{{\rm{W}}}_{{\rm{C}}}\,\ast \,{{\rm{R}}}_{13{\rm{C}}}/{{\rm{R}}}_{12{\rm{C}}}}{1+{{\rm{R}}}_{13{\rm{C}}}/{{\rm{R}}}_{12{\rm{C}}}}$$

3 $${{\rm{M}}}_{13{\rm{c}}}={{\rm{M}}}_{{\rm{c}}}\,\ast \,{{\rm{R}}}_{13C}$$

where R_13C_ and R_12C_ are the percentages of ^13^C and ^12^C in the soil, respectively, in units of %, W_c_ is the content of total organic C in the tested soil sample in %, M_c_ is the content of a specific SOC fraction in mg kg^−1^, and M_13c_ is the ^13^C-SOC content of a specific SOC fraction in mg kg^−1^.

To determine the contribution of straw-C to SOC and SOC fractions, the contribution rate was calculated as follows:

4 $${{\rm{F}}}_{{\rm{m}}}=({\delta }^{13}{{\rm{C}}}_{{\rm{sm}}}-{\delta }^{13}{{\rm{C}}}_{{\rm{s}}})/({\delta }^{13}{{\rm{C}}}_{{\rm{m}}}-{\delta }^{13}{{\rm{C}}}_{{\rm{s}}})\times 100,$$

where F_m_ is the contribution rate of straw-C to SOC or SOC fractions in units of %, δ^13^C_sm_ is the δ^13^C value of the tested soil with straw inputs in ‰, δ^13^C_s_ is the δ^13^C value of the tested soil without straw inputs in ‰, and δ^13^C_m_ is the δ^13^C value of the experimental straws in ‰.

## Results

### Transformation and distribution of straw-C to SOC fractions

**Figure 1:**
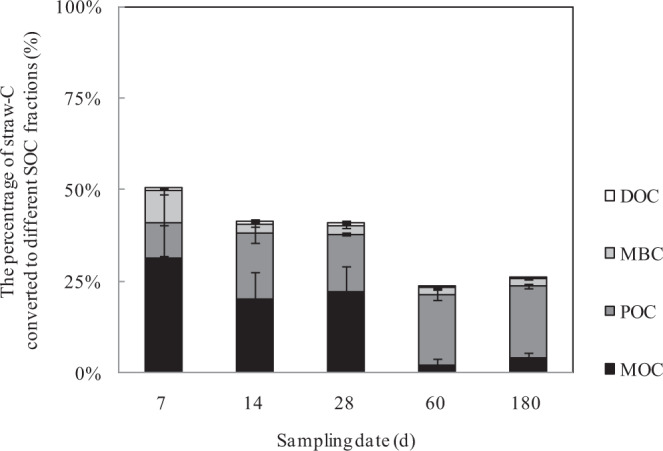
Straw-C transformation and distribution to various SOC fractions.

We assumed that the selection difference between the ^12^C and ^13^C by each organic C pool was negligible, and ignored the difference of the distribution of labeled ^13^C in different parts of straw. Thus, the proportion of increased ^13^C-SOC fractions in the total ^13^C of the straw was equal to the percentage of straw-C converted to various SOC fractions (DOC, MBC, POC and MaOC). The results showed that the maximum straw-C conversion rate of 50.7% was obtained on day 7 after straw inputs, after which the conversion rates decreased to 26.0% on day 180 (Fig. 1). The distribution of straw-C to the different SOC fractions changed with time. On day 7, about 31.4% of straw-C was transformed to soil MaOC, and 9.75% and 8.50% of straw-C had been converted into soil POC and MBC, respectively, but only 0.99% of straw-C had converted to soil DOC. The content of straw-C converted to soil DOC varied in the range of 1.06–0.91% during days 14–28, and further declined to close to 0.0% after 60 days. Similarly, the conversion ratio of straw-C to soil MaOC varied in the range of 20.08–22.33% during days 14–28, and reduced sharply to 2.26% on day of 60. But it presented a slight increase from day of 60 to day of 180 with the conversion ratio of 3.93%. Compared with DOC and MaOC, the conversion ratio of straw C to soil MBC maintained relatively stable after day 7 in the range of 2.22–2.28%, while the conversion ratio of straw-C to soil POC increased slightly with time, ranging from 15.6% to 19.8%.

### Contribution of straw-C to SOC

**Figure 2:**
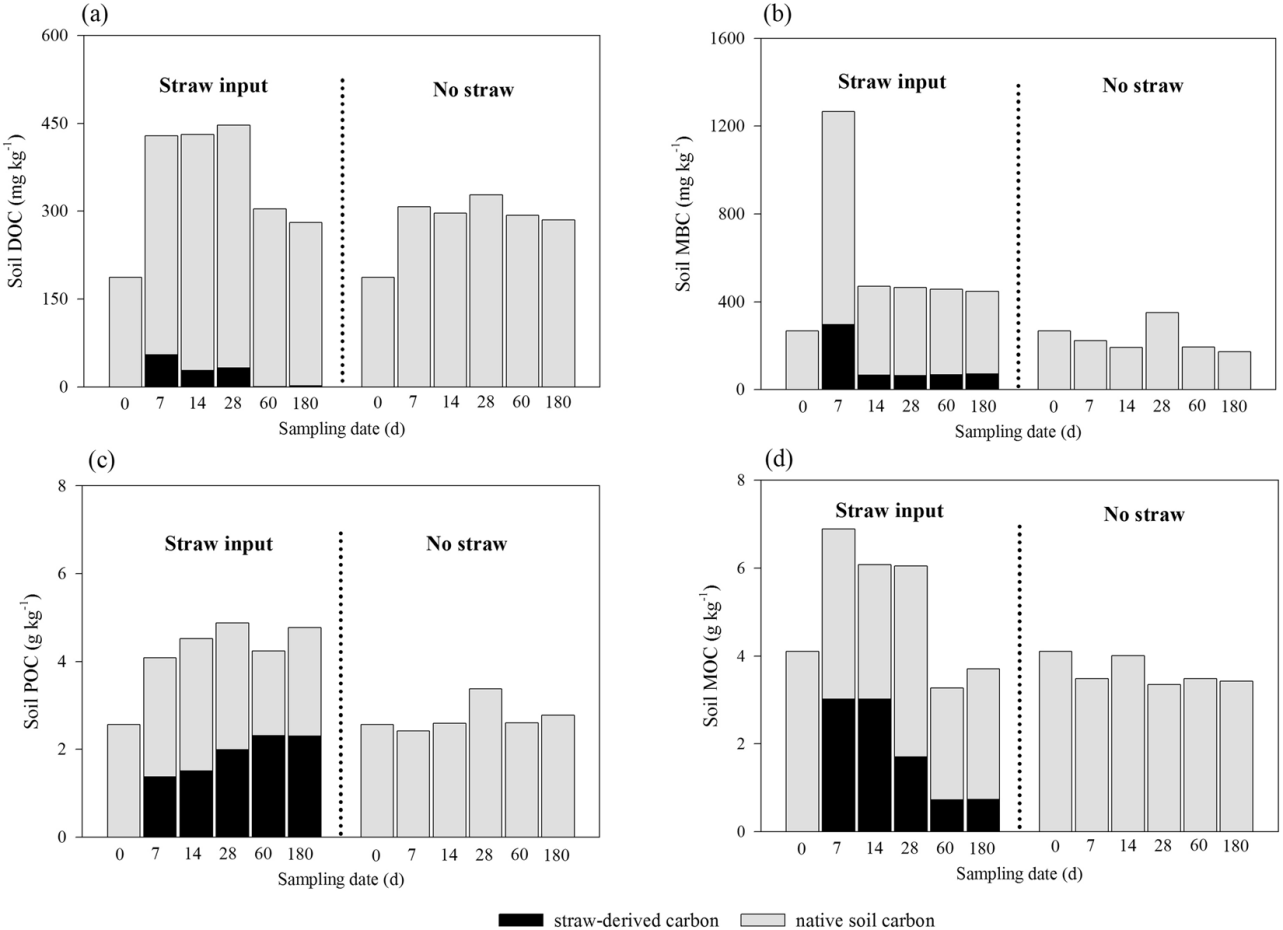
The effect of straw inputs on the contents of soil (**a**) DOC, (**b**) MBC, (**c**) POC and (**d**) MaOC, and the contribution of straw-C (black bar) and native soil carbon (gray bar) to these SOC fractions in the first 180 days of straw decomposition.

During the straw C transformation and distribution process, the contents of soil DOC, MBC, POC and MaOC changed with time (Fig. 2). Within the first 7 days after straw inputs, soil DOC, MBC, POC and MaOC contents had the largest increase, which was 0.39, 4.69, 0.69 and 0.98 times higher than that of the control, respectively. The DOC content varied in the range of 430.7–447.0 mg kg^−1^ between days 14 and 28, and decreased to 280.6–303.6 mg kg^−1^ after 60 days, which was non-significantly different from the control (*P*_*60 days*_ and *P*_*180 days*_ > 0.05, Fig. 2a). The content of soil MBC decreased dramatically after 7 days with straw inputs and maintained at 447.4–470.3 mg kg^−1^ from days 14 to 180, which was 0.3–1.6 times higher than that of the control (Fig. 2b). Straw-C contribution also peaked on day 7 after straw inputs for soil DOC (13.0%) and MBC (23.54%). The soil POC content after straw inputs increased with the time throughout the entire incubation period, while the soil MaOC content decreased during days 14 to 60, and increased slightly to about 3.71 g kg^−1^ on day 180 (Fig. 2c,d). The contribution rate of straw-C to soil POC increased from 33.94% on day 7 to 54.7% on day 60 after straw inputs, but decreased to 48.6% on day 180. For soil MaOC, straw-C contributed about 44.0% and 49.9% on average on day 7 and 14 after straw inputs, respectively, and then the straw-C contribution decreased with time, with only 20.0% of MaOC derived from straw on day 180 after straw inputs. However, the estimated effects of ^13^C-labaled straw inputs on SOC fractions might be conservative because of our hypotheses that there exists no discrimination effect between ^12^C and ^13^C and no different distribution of labeled ^13^C in experimental straw.

### Dynamics of diversity, structure, and taxonomic composition of soil bacterial communities

**Figure 3:**
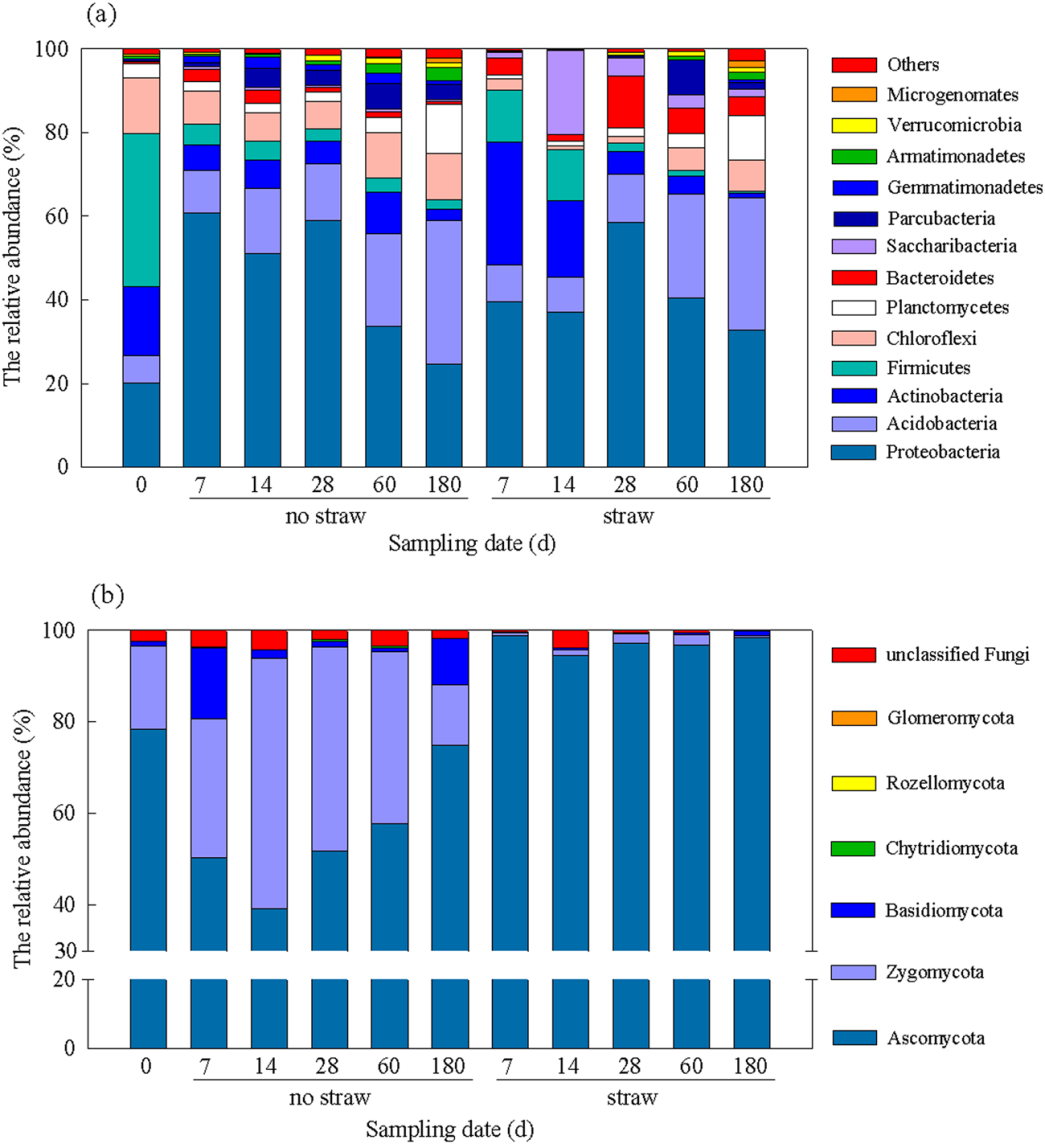
The dominant bacteria (**a**) and fungi (**b**) phyla with and without straw inputs during the 180 day of incubation.

**Figure 4:**
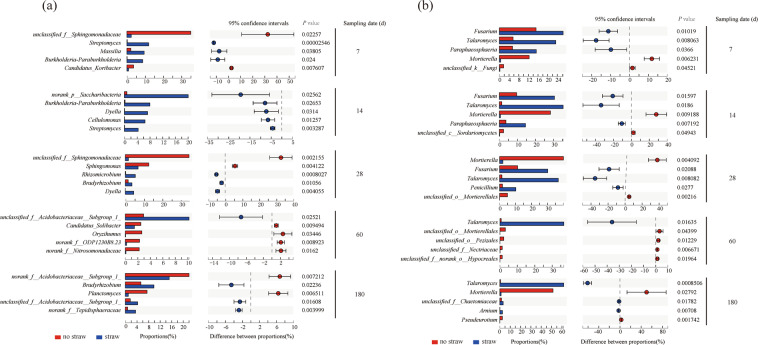
Pairwise comparison of the dominant genera in the bacterial (**a**) and fungal (**b**) communities between treatments with and without straw inputs on days 7, 14, 28, 60, and 180. Bars in blue and red represent the treatment with and without straw inputs, respectively.

Soil bacterial community diversity indexes were calculated based on the OTU data from the Miseq sequencing results shown in Fig. S1(a). There was a significant difference between the treatments with and without straw inputs only on day 60, while NMDS analysis showed that the soil bacterial community structure changed significantly after straw inputs (Fig. S2a). Compared with the control, the relative abundance of Proteobacteria decreased slightly and Firmicutes and Actinobacteria increased greatly in the initial phase of straw-C conversion (the first 7–14 days), after which the relative abundance of Proteobacteria, Bacteroidetes, and Acidobacteria increased, but Firmicutes and Actinobacteria decreased (Fig. 3a). On the genus level, the relative abundance of *Streptomyces*, *Burkholderia-Paraburkholderia*, and *Massilia* increased to their maxima on day 7 of 11.9%, 8.6%, and 9.4%, respectively, which were significantly higher than the control (Figs. 4a and S3a). On day 14, the genus of norank-p*-Saccharibacteria*, *Dyella*, and *Cellulomonas* increased to their maximums at 20.1%, 7.3%, and 6.5%, respectively, all of which were significantly higher than the control, and then decreased with time. During the later phase of straw-C decomposition, the major genera with a significantly higher abundance belonged to *Rhizomicrobium*, *Bradyhizobium*, *Dyella*, and unclassified *Acidobacteriaceas*-subgroup-1(Fig. 4a).

### Dynamics of diversity, structure, and taxonomic composition of soil fungal communities

The Shannon diversity indexes of the soil fungal communities decreased with straw inputs to 1.79–2.60 for the duration of the incubation, while the Shannon indexes without straw inputs was 2.83–3.46 (Fig. S1b). NMDS analysis showed that the soil fungal community structure changed significantly after straw inputs for the duration of the incubation (Fig. S2b). Compared with the control, there was a large increase in the phylum Ascomycota with straw inputs from the initial to the late phase of straw decomposition (Fig. 3b), with a relative abundance of 94.4–98.9%. On the genus level, the relative abundance of *Talaromyces* increased with time and reached 61.6% on day 180 after straw inputs (Figs. 4b and S3b). Straw inputs also significantly increased the abundance of *Fusarium*, *Paraphaeosphaeria*, *Penicillium*, unclassified *Chaetomiaceae*, and *Arnium* at different phases of straw decomposition (Fig. 4b).

### Statistically significant microbial communities during the whole incubation

**Figure 5:**
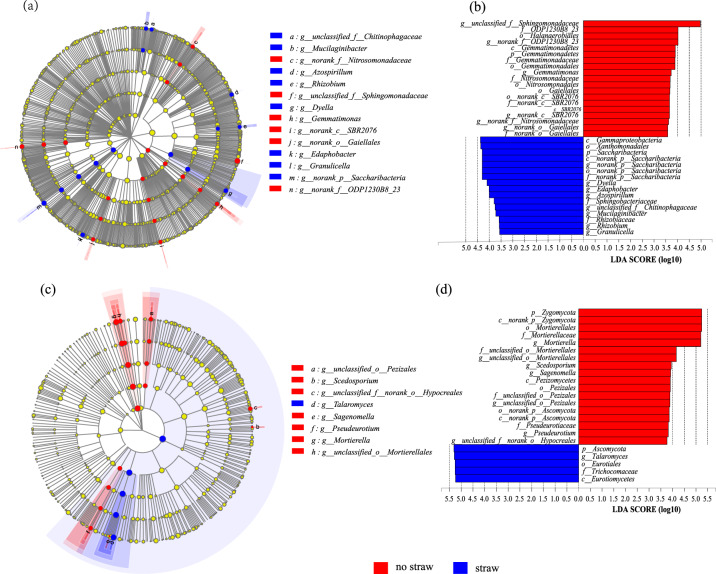
Linear discriminative analysis (LDA) effect size LEfSe analysis of the bacterial (**a**,**b**) and fungal (**c**,**d**) genera between the treatment with (blue) and without (red) straw inputs. LDA scores (log10) for the most discriminative genera in the soils with straw inputs were represented on the negative scale; positive LDA scores indicated the enriched genera in the soil without straw inputs. Bars in blue and red represent the treatment with and without straw inputs, respectively.

LEfSe analysis was applied from the domain to the genus level in the study to identify specialized communities in samples. The groups with LDA scores of 3.5 or greater were shown in cladograms, and were confirmed by LEfSe (Fig. 5). The result showed that eight groups of bacteria and one group of fungi were significantly enriched in the treatment with straw inputs during the whole incubation, namely the *Dyella*, *Mucilaginibacter*, *Rhizobium*, norank-p-*Saccharibacteria*, *Azospirillum*, *Edaphobacter*, *Granulicella*, and unclassified *Chitinophagaceae* genera (Fig. 5a) and the genus *Talaromyces* (Fig. 5c).

### Soil microbial communities are linked to straw-C conversion

The soil microbial community distance matrices correlated significantly with SOC fractions derived from the straw (R_bacteria_ = 0.636, *p*_bacteria_ = 0.001,R_fungi_ = 0.689, *p*_fungi_ = 0.003) and SOC fraction distance matrices (R_bacteria_ = 0.306, *p*_bacteria_ = 0.035, R_fungi_ = 0.538, *p*_fungi_ = 0.005) in the treatment with straw inputs. However, we observed no significant correlation between the soil microbial community and SOC fraction distance matrices in the control treatment (R_bacteria_ = −0.126, *p*_bacteria_ = 0.441, R_fungi_ = 0.120, *p*_fungi_ = 0.512). This suggests that the soil bacteria and fungi govern the straw-C transformation and distribution, and further regulate SOC change after straw inputs.

**Figure 6:**
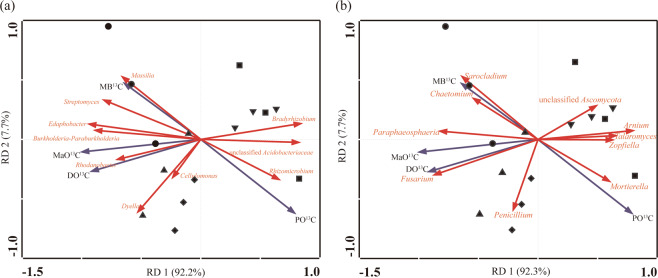
Redundancy discriminate analysis of soil bacterial (**a**) and fungal (**b**) communities and SOC fractions derived from straw-C after straw inputs. The symbols ●, ▲, ◆, ■ and ▼ represent the soils sampled on days 7, 14, 28, 60, and 180 after straw inputs, respectively.

Redundancy analysis showed that the coordinates from the first two ordination axes explained more than 90.0% of the variance of straw-C distribution to SOC (Fig. 6). A Monte Carlo permutation test showed that the bacteria *Streptomyces*, *Massilia*, *Sphingobacterium*, *Edaphobacter*, *Dyella*, *Burkhoderia-Paraburkholderia*, unclassified *Acidobacteriaceae*, and *Bradyrhizobium*, and the fungi *Talaromyces*, *Fusarium*, *Paraphaeosphaeria*, and unclassified *Sordariomycetes* were significantly correlated with straw-C distribution to SOC (*P* < 0.05).

We analyzed the significant genera and each straw-derived SOC fraction using Spearman correlations, as reported in Table 1. We found that straw-derived DOC and MaOC were positively correlated with the bacteria *Streptomyces*, *Edaphobacter*, *Burkholderia-Paraburkholderia*. POC-derived from straw was positively correlated with the fungi *Talaromyces*, the bacteria unclassified *Acidobacteriaceae*, *Bradyrhizobium*, and unclassified *Sordariomycetes* (R > 0.5, *P* < 0.05), which were negatively correlated with straw-derived DOC and MaOC (R < −0.6, *P* < 0.01). Straw-derived MBC was positively correlated with the bacteria *Massilia* and *Streptomyces* (R > 0.9).

**Table 1:** Correlation coefficient (R value) of Spearman correlations for specific genus and SOC fractions derived from decomposing straw.

Function type	Genus	SOC fractions derived from straw			
		DOC	MaOC	POC	MBC
Copiotrophic	*Massilia*	0.393	0.692**	−0.820**	0.979**
	*Streptomyces*	0.627*	0.811**	−0.773**	0.937**
Cellulatic	*Fusarium*	0.837**	0.790**	−0.449	0.256
	*Dyella*	0.727**	0.441	0.043	−0.239
	*Talaromyces*	−0.684**	−0.703**	0.590*	−0.593*
Acidophilic	*Edaphobacter*	0.803**	0.890**	−0.709**	0.752*
	unclassified *Acidobacteriaceae*	−0.656**	−0.755**	0.515**	−0.543*
Denitrifying	*Burkholderia-Paraburkholderia*	0.803**	0.839**	−0.660**	0.602*
	*Paraphaeosphaeria*	0.758**	0.738**	−0.487	0.641*
N−fixing	*Bradyrhizobium*	−0.867**	−0.838**	0.595**	−0.556*
Others	unclassified *Sordariomycetes*	−0.902**	−0.874**	0.561*	−0.483

^*^P < 0.05; **P < 0.01; ***P < 0.001.

## Discussion

In this study, we investigated the effects of straw inputs on soil bacterial and fungal communities and their relationship to straw-C distribution to SOC fractions during the first 180 days of straw decomposition. Our results supported our hypotheses that straw inputs significantly changed the soil bacterial and fungal communities between the initial and late phases of straw decomposition. We also found evidence that soil microbial communities, including copiotrophic, cellulatic, acidophilic and certain N-fixing and denitrifying microbes, were positively correlated with straw-C distributions.

Straw-C was rapidly transformed and distributed into different SOC forms by soil microorganisms in the initial 7 days after straw input. We mainly attributed this to fast-growing populations, including phyla like Proteobacteria, Firmicutes, Actinomycetes, and Ascomycota, that can rapidly take advantage of the presence of degradable C compounds in the wheat straw and other C-rich environments^37^. Pascault *et al*.^24^ and Fan *et al*.^25^ also reported that these phyla were the dominant bacteria assimilating C derived from wheat or maize straws in the initial phase of straw decomposition. On the genus level, the relative abundance of the bacteria *Streptomyce* and *Massilia*, and the fungi *Talaromyces* and *Fusarium*, increased significantly during the initial phase of straw decomposition in our experiment. The *Streptomyce* and *Massilia* genera have been previously described as consisting of mainly copiotrophic bacteria^37^, which might be more competitive than others in utilizing DOC derived from straw C, such as sugar, saccharose, fructose and amino acid, in the initial phase of straw decomposition. Such labile organic C represented most of straw-C. Most of these C compounds are generally associated with higher CUE values, which can be utilized by microbes as C and energy source to biosynthesize and growth^13,25^. This might be the main reason for the large increase of MBC and straw contribution on day 7 after straw inputs and this also explained the positive correlation between the genera and MBC-derived from straw. *Fusarium* has been reported to have a strong ability to produce a variety of lignocellulosic enzymes with synergistic effects on lignocellulosic hydrolysis^38^. Significantly positive correlations between *Streptomyce*, *Massilia*, *Fusarium* and straw-derived DOC and/or MaOC in our results indicated that these microbes might be involved in the transformation and distribution of straw-C to soil DOC as well as the formation of MaOC. As is known, during the process of lignocellulosic hydrolysis, intermediate products such as monosaccharides, amino acid, low-molecular-weight organic acid and short-chain fatty acids can be produced. Following such C compounds assimilation and microbial biosynthesis, then their death and turnover can directly contribute to the MaOC pool^13^. This explained the relatively higher ratio of straw-C converted to MaOC on day 7 after straw inputs, which was also consist with the results reported by Lavallee^39^, who observed a large increase in the amount of litter derived C stabilized in a short time (6–9 days) with a 13% and 30% increase in %C in the clay and silt fraction, respectively. *Talaromyces* has been demonstrated to have a cellulolytic system^40^, and had a significant positive correlation with POC-derived from straw in the study (R = 0.590, *P* < 0.05). It is speculated that this genus may be related to certain organic C components in the process of straw-C conversion, such as cellulose, hemicellulose and lignin, which can enter soil POC. Notably, the bacteria *Burkholderia-paraburkholderia* and the fungi *Paraphaeosphaeria* were also significantly increased on 7 days following straw inputs and had positive correlations with DOC-, and MaOC-derived from straw. The two genera are both capable of soil denitrification^40,41^ and can utilize soil available organic C as their C source to support their growth and then might contribute to MaOC formation through *in vivo* microbial turnover pathway^12^. Fan *et al*.^25^ applied DNA-SIP to detect the bacteria assimilating C from maize residues and also observed the existence of nitrite-reducing taxa. The decomposition of straw might lead to transitly anoxic conditions, which were suitable for the growth of denitrifying microbes. This might be another important reason for denitrifying populations increase during a decomposition process.

After the initial 7 days of straw decomposition, more cellulolytic bacteria such as *Dyella*^30^ and *Cellulomonas* increased. Positive correlations between *Dyella* and DOC-derived from straw indicated that this genus became the dominant driver for the further transformation of straw C into soil DOC in the later phases of straw decomposition. *Acidobacteria* is a large phylum which include many classes not sensitive to soil pH and is also described as a phylum composed by mainly oligotrophic and slow growing population. This might be the reason for the increase of unclassified *Acidobaceriaceae* and *Edaphobacter* after the copiotrophic taxa. Interestingly, the opposite correlation with MaOC-derived from straw was observed with *Acidobaceriaceae* and *Edaphobacter*. We speculated that the carbon source preferences of these two genera might be different. After initial straw decomposition, the mineral-associated C compounds with weaker sorption (e.g., amino acid, short-chain fatty acid) might be further desorbed and then be assimilated by microbes, such as unclassified *Acidobaceriaceae*, which had a significantly negative correlation with MaOC derived from straw. The sharp decrease of MaOC from days 14 to 60 may be the result of desorption after the saturation of organic C in mineral fraction, due to the relatively low clay contents of the experimental soil that provided limited mineral-surface^21^. On the contrary, the genus of *Edaphobacter* might utilize straw/microbial-derived DOC to grow during the whole phase, which byproducts can be absorbed in mineral surfaces and thus benefit to MaOC formation.

Additionally, at this phase, more straw derived-C, mainly of structural polymers, such as lignin, accumulated and formed POC, positively correlated with *Bradyrhizobium*, which presented a significant increase during the later straw decomposition phase. *Bradyrhizobium* has been reported to accumulate poly-3-hydroxybutyrate (PHB) in the cell under C-rich conditions and utilize PHB as its C and energy source when the content of available C in soils decreased^42^. Thus, we speculated that after the initial phase of straw decomposition, most of the available organic C had been utilized and many microbes decreased (see Fig. 2b) due to limited available C sources, but *Bradyrhizobium* can utilize PHB stored in its cells as a source of C and energy to support growth. Moreover, *Bradyrhizobium* can secrete extracellular polymeric substances (EPS) in response to environmental stresses, e.g., pH, temperature, carbon source shortage and toxic components^43,44^. EPS can promote the formation of soil aggregates through electrostatic action, intermolecular van der Waals force or adsorption^45^, and thus improve the distribution of straw C to POC and MaOC. This might be the main reason for the significant positive correlation we observed between the abundance of *Bradyrhizobium* and POC-derived from straw, and explained the slight increase in MaOC from the day 60 to 180 after straw inputs. This link suggests that *Bradyrhizobium* played an important role in straw-C distribution to SOC in later straw decomposition phase.

The key genera enhanced during the whole 180 days of straw decomposition included cellulolytic bacteria (*Dyella*, unclassified *Chitinophagaceae*)^46^ and fungi (*Talaromyces*), N-fixing bacteria (*Rhizobium* and *Azospirillum*), acidophilic bacteria (*Edaphobacter* and *Granulicella*)^47^, and others including *Mucilaginibacter*, norank-p-*Saccharibacteria* that are capable of degrading pectin, xylan, glucose, amino acids, and some other polysaccharides^48,49^. Although some of these organisms had relatively lower abundance and no significant effect on straw C distribution to SOC, they may play an important role in coordinating the decomposition of straw. Like *Bradyrhizobium*, *Rhizobium* can also synthesize and accumulate PHB and utilize it under C-limited conditions^50–52^, which might be the main reason for its consistent increase throughout the incubation. It indicated that the bacteria having alternative ways to use/store/access C sources derived from straw-C, could easily increase throughout the incubation period and then promote soil C sequestration. *Azospirillum* has been identified as a genus that can fix N symbiotically and independently^53–55^. Thus, the promotion of *Azospirillum* after straw inputs may in-turn provide available N into the soil system that could support soil microbes in order to complete the spectrum of straw C transformations.

**Figure 7:**
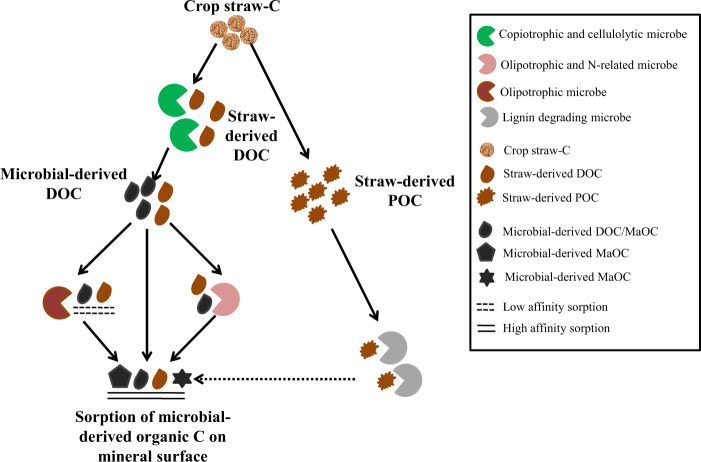
The conceptual scheme of the straw decomposition with the succession of microbial populations and their probable role in the fractionation of the straw-derived C. After straw inputs, amount of DOC () is derived from straw-C (), and can be firstly utilized by fast-growing and cellulolytic microbes (), who will produce microbial-derived organic C compounds (). Part of these C compounds can be directly absorbed on mineral surfaces with relatively lower () or higher sorptive affinity (), while some of the adsorbed C with relatively lower sorptive affinity might be desorbed and further be degraded by certain olipotrophic microbes () (e.g., unclassified *Acidobacteriaceae*) in the following phase. The other part can be utilized directly by certain microbes (), such as slow-growing microbe (e.g. *Edaphobacter*) and N-related microbe (e.g. *Burkholderia-Paraburkholderia* and *Bradyrhizobium*). The byproducts C () (e.g., aromatic C, phenolic acids, extracellular polymeric substances) from the above microbes will be adsorbed in mineral surface with relatively higher sorptive affinity () and contributed to MaOC. During the whole process of straw decomposition, the structural straw C, such as lignin and hemicellulose, accumulated and formed POC (). But this part of POC may be also decomposed by microbes () in long-term, and the microbial-derived C from them () might absorb in mineral surface (). However, this link has not been confirmed in this study, thus it is depicted as dotted arrow.

Based on these results, we proposed a conceptual scheme of the straw decomposition with the succession of microbial populations and their role in the fractionation of the straw-derived C, shown in Fig. 7. Generally, after straw inputs, fast-growing and cellulolytic microbes are quickly involved in the straw-C conversion and produced microbial-derived DOC. Part of straw-derived DOC and microbial-derived DOC can be directly absorbed on mineral surfaces with low or high sorptive affinity, and some C absorbed on mineral surfaces with low sorptive affinity might be desorbed and further be decomposed by certain slow-growing bacteria, e.g. unclassified *Acidobacteriaceae*, while the other part of DOC can be utilized by specific microbes directly in the later phase, such as acidopilic and denitrifying microbes, *Edaphobacter* and *Bradyrhizobium*. The byproducts C (e.g., aromatic C, phenolic acids) from all these microbes will be adsorbed in mineral surface with relatively higher sorptive affinity, contributing to soil MaOC. During the whole process of straw decomposition, the structural straw C, such as lignin and hemicellulose, accumulated and formed POC. But this part of POC may be also degraded by microbes in long-term, and the microbial-derived C from them might absorb in mineral surface, which needs further study by constructing a long-term experiment.

## Conclusion

Our results showed that the soil microbial community in soils with straw inputs changed significantly over the first 180 days after straw inputs. This temporal change of soil microbial community structure well reflected the conversion and distribution process of straw-C to different SOC fractions. Copiotrophic (e.g., *Massilia* and *Streptomyces*), cellulolytic (*Fusarium and Dyella*), acidophilic (*unclassified Acidobaceriaceae* and *Edaphobacter*) and certain microbes with specific function (e.g., denitrifying and N-fixing) played important role in different SOC fraction formation and dynamic change during the straw decomposition process. However, conducting a long-term experiment is necessary to further study the role of soil microbes in promoting MaOC accumulation and/or POC transformation, which helps to better understand how straw return affect soil C sequestration in a longer time schedule.

## Supplementary information

Supplementary Figures
