# Author Correction: Linking soil microbial community dynamics to straw-carbon distribution in soil organic carbon

**DOI:** 10.1038/s41598-020-68527-9

**Published:** 2020-07-07

**Authors:** Yao Su, Zhenchao He, Yanhua Yang, Shengqiang Jia, Man Yu, Xijing Chen, Alin Shen

**Affiliations:** 1 Institute of Environment, Resource, Soil and Fertilizer, Zhejiang Academy of Agricultural Sciences, Hangzhou, 310021 China; 2 College of Environment and Resources, Zhejiang A & F University, Hangzhou, 311300 China

Correction to: *Scientific Reports* https://doi.org/10.1038/s41598-020-62198-2, published online 26 March 2020

This Article contains a typographical error in the Acknowledgements section.

“This work was financially supported by the China Natural Science Foundation Youth Fund Project (41807034), Zhejiang Natural Science Fund Youth Fund Project (LQ18030001), and the National Modern Agricultural Technology System of Wheat (CARS-3).The authors would like to thank professor Fanqiao Meng’s team from China Agricultural University for providing us with the labeled wheat straws samples.”

should read:

“This work was financially supported by the China Natural Science Foundation Youth Fund Project (41807034), Zhejiang Natural Science Fund Youth Fund Project (LQ18D030001) and the National Modern Agricultural Technology System of Wheat (CARS-3). The authors would like to thank professor Fanqiao Meng’s team from China Agricultural University for providing us with the labeled wheat straws samples.”

