# Supplementary material for: Linking soil microbial community dynamics to straw-carbon distribution in soil organic carbon
Source: Sci Rep. 2020 Mar 26;10:5526. doi: 10.1038/s41598-020-62198-2 (PMC7099027; doi:10.1038/s41598-020-62198-2)
Supplement: Supplementary file 1 — Supplementary Figures [file 41598_2020_62198_MOESM1_ESM.pdf]

Supplementary material

Linking soil microbial community dynamics to straw-carbon distribution in soil organic carbon

Yao Su[1], Zhenchao He[1], Yanhua Yang[1,2], Shengqiang Jia[1,2], Man Yu[1], Xijing Chen[1], Alin Shen[11]

[1]*Institute of Environment, Resource, Soil and Fertilizer, Zhejiang Academy of Agricultural Sciences, Hangzhou 310021, China*

[2]*College of Environment and Resources, Zhejiang A & F University, Hangzhou 311300, China*

[1]Corresponding author. Tel.:+86 571 88166275; Fax: +86 571 88166275;

Email: shenalin_aee@163.com

Figure captions

Fig. S1 The Shannon index of the soil bacterial (a) and fungal (d) community. Bars in blue and red represent the treatment with and without straw inputs, respectively.

Fig. S2 Comparison of temporal changes of the soil bacterial (a) and fungal (b) community structures with and without straw inputs during the soil incubation via non-metric multidimensional scaling (NMDS) analysis. The ordinations were based on a Bray-Curtis distance method. Marks in blue and red represent the treatment with and without straw inputs, respectively.

Fig. S3 The dominant bacteria (a) and fungi (b) genera with and without straw inputs during the 180 day of incubation.

Fig. S1

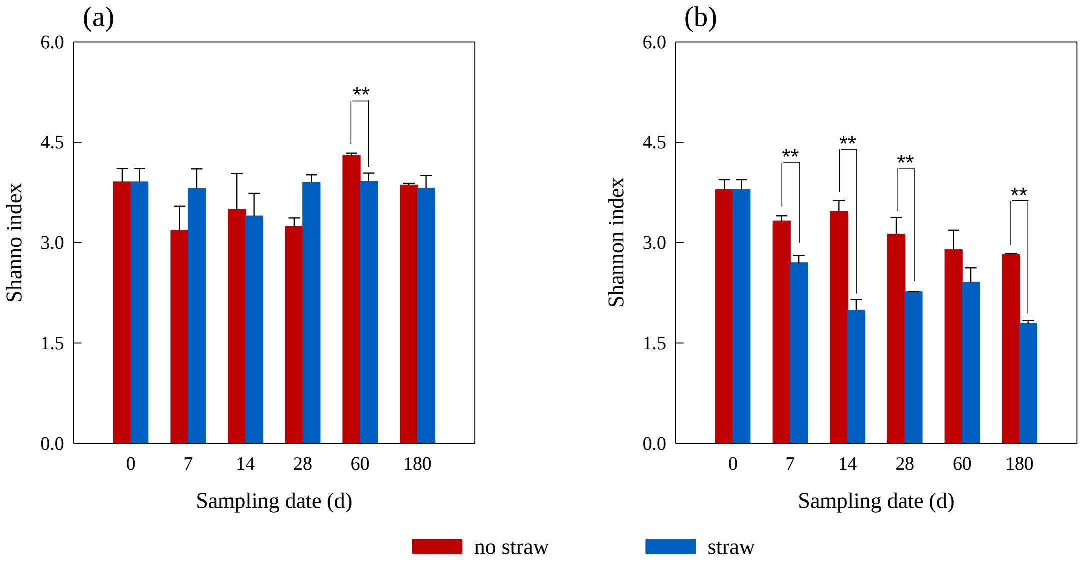
(a)
6.0
4.5
3.0
1.5
0.0
Shanno index
**
0
7
14
28
60
180
Sampling date (d)
(b)
6.0
4.5
3.0
1.5
0.0
Shannon index
**
**
**
**
0
7
14
28
60
180
Sampling date (d)
no straw
straw

Fig. S2

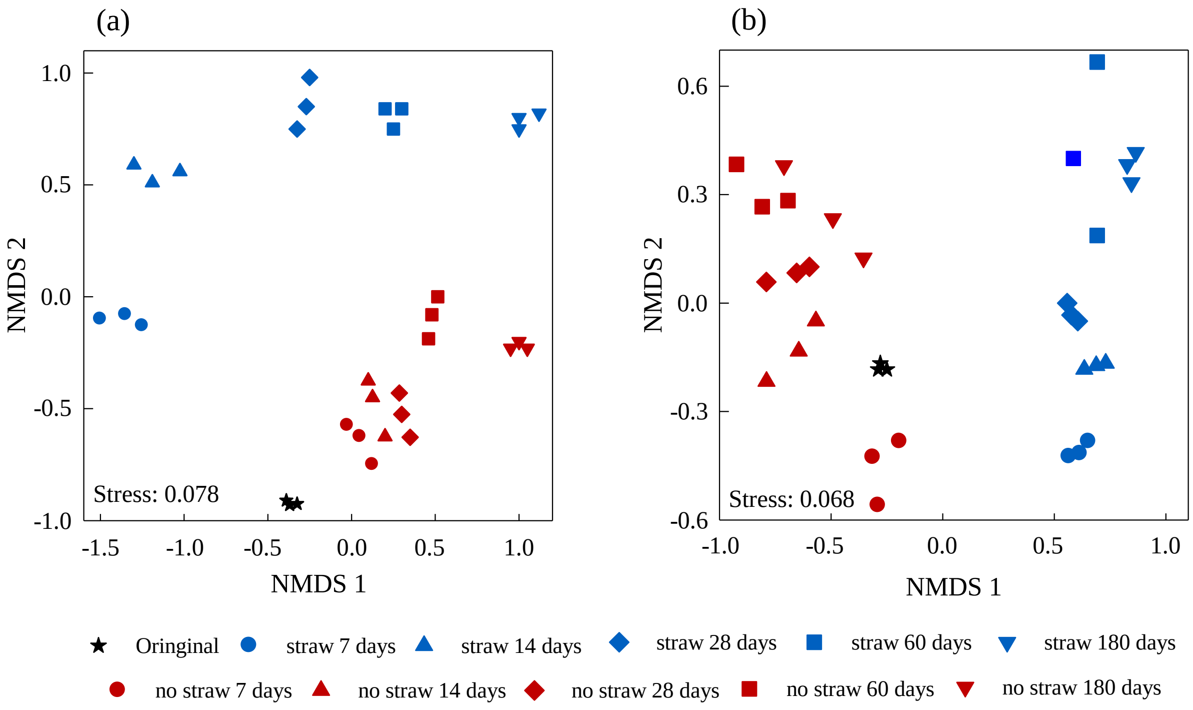
(a)
(b)
NMDS 2
NMDS 1
Stress: 0.078
Stress: 0.068
1.0
0.5
0.0
-0.5
-1.0
-1.5
-1.0
-0.5
0.0
0.5
1.0
0.6
0.3
0.0
-0.3
-0.6
Oringinal
straw 7 days
straw 14 days
straw 28 days
straw 60 days
straw 180 days
no straw 7 days
no straw 14 days
no straw 28 days
no straw 60 days
no straw 180 days

Fig. S3

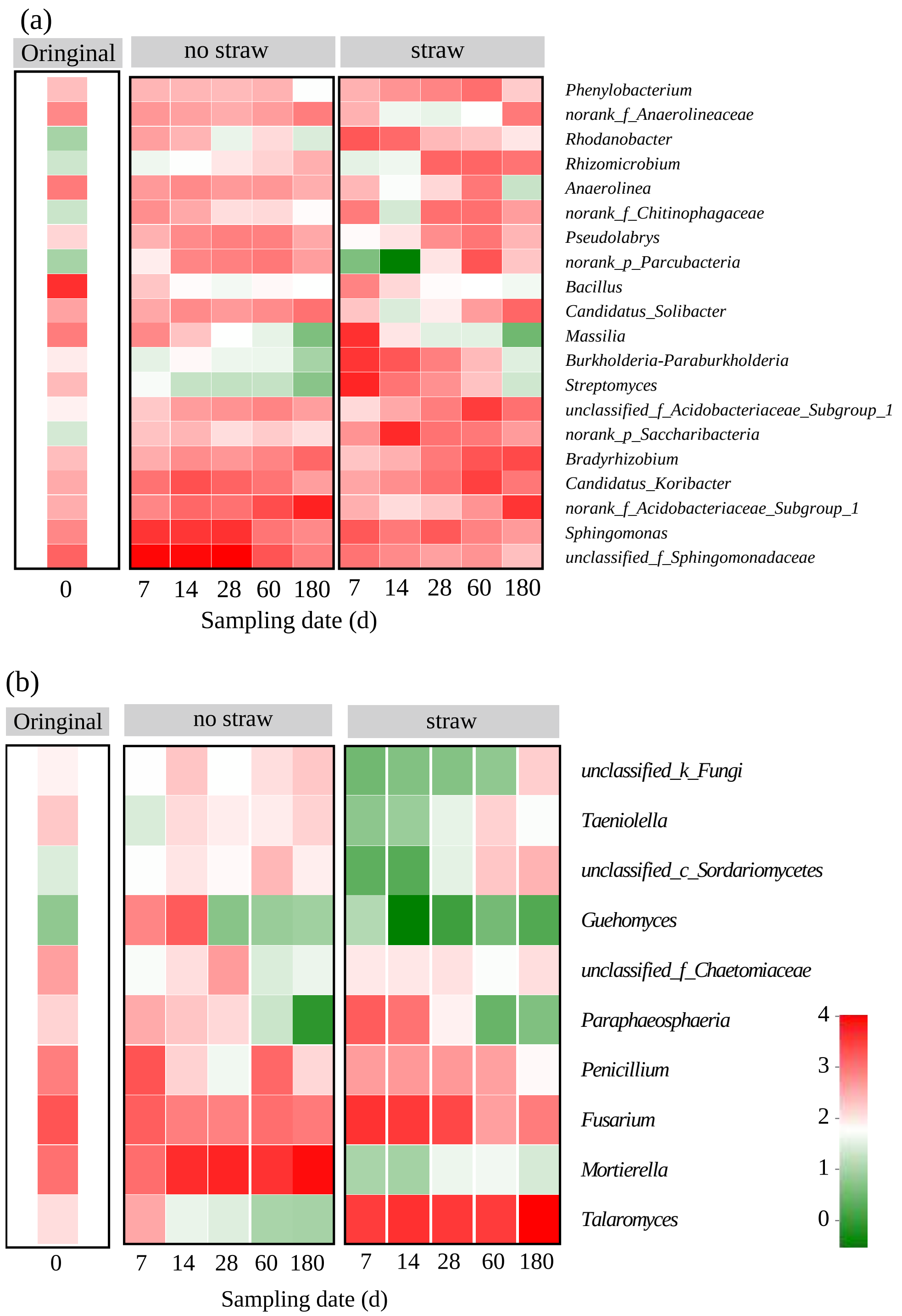
(a)
Oringinal
no straw
straw
Phenylobacterium
norank_f_Anaerolineaceae
Rhodanobacter
Rhizomicrobium
Anaerolinea
norank_f_Chitinophagaceae
Pseudolabrys
norank_p_Parcubacteria
Bacillus
Candidatus_Solibacter
Massilia
Burkholderia-Paraburkholderia
Streptomyces
unclassified_f_Acidobacteriaceae_Subgroup_1
norank_p_Saccharibacteria
Bradyrhizobium
Candidatus_Koribacter
norank_f_Acidobacteriaceae_Subgroup_1
Sphingomonas
unclassified_f_Sphingomonadaceae
0
7 14 28 60 180
7 14 28 60 180
Sampling date (d)
(b)
Oringinal
no straw
straw
unclassified_k_Fungi
Taeniolella
unclassified_c_Sordariomycetes
Guehomyces
unclassified_f_Chaetomiaceae
Paraphaeosphaeria
Penicillium
Fusarium
Mortierella
Talaromyces
4
3
2
1
0
0
7 14 28 60 180
7 14 28 60 180
Sampling date (d)
